# Intravenous Thrombolysis May Not Improve Clinical Outcome of Acute Ischemic Stroke Patients Without a Baseline Vessel Occlusion

**DOI:** 10.3389/fneur.2018.00405

**Published:** 2018-06-06

**Authors:** Huiqiao Tian, Mark W. Parsons, Christopher R. Levi, Xin Cheng, Richard I. Aviv, Neil J. Spratt, Timothy J. Kleinig, Billy O'Brien, Kenneth S. Butcher, Longting Lin, Jingfen Zhang, Qiang Dong, Chushuang Chen, Andrew Bivard

**Affiliations:** ^1^Department of Neurology, John Hunter Hospital, University of Newcastle, Newcastle, NSW, Australia; ^2^Department of Neurology, Huashan Hospital, Fudan University, Shanghai, China; ^3^Division of Neuroradiology, Department of Medical Imaging, University of Toronto and Sunnybrook Health Sciences Centre, Toronto, ON, Canada; ^4^Department of Neurology, Royal Adelaide Hospital, Adelaide, SA, Australia; ^5^Department of Neurology, Gosford Hospital, Gosford, NSW, Australia; ^6^Division of Neurology, Department of Medicine, University of Alberta, Edmonton, AB, Canada; ^7^Department of Neurology, Baotou Central Hospital, Baotou, China

**Keywords:** acute ischemic stroke, alteplase, vessel patency status, perfusion lesion, CT perfusion

## Abstract

**Background and Purpose:** The benefit of thrombolysis in ischemic stroke patients without a visible vessel occlusion still requires investigation. This study tested the hypothesis that non-lacunar stroke patients with no visible vessel occlusion on baseline imaging would have a favorable outcome regardless of treatment with alteplase.

**Methods:** We utilized a prospectively collected registry of ischemic stroke patients [the International Stroke Perfusion Imaging Registry (INSPIRE)] who had baseline computed tomographic perfusion and computed tomographic angiography. The rates of patients achieving modified Rankin Scale (mRS) 0–1 were compared between alteplase treated and untreated patients using logistic regression to generate odds ratios.

**Results:** Of 1569 patients in the INSPIRE registry, 1,277 were eligible for inclusion. Of these, 306 (24%) had no identifiable occlusion and were eligible for alteplase, with 141 (46%) of these patients receiving thrombolysis. The treated and untreated groups had significantly different median baseline National Institutes of Health Stroke Scale (NIHSS) [alteplase 8, interquartile range (IQR) 5–10, untreated 6, IQR 4–8, *P* < 0.001] and median volume of baseline perfusion lesion [alteplase 5.6 mL, IQR 1.3–17.7 mL, untreated 2.6 mL, IQR 0–6.7 mL, *P* < 0.001]. After propensity analysis, alteplase treated patients without a vessel occlusion were less likely to have an excellent outcome (mRS 0–1; 56%) than untreated (78.8%, OR, 0.42, 95% confidence interval, 0.24–0.75, *P* = 0.003).

**Conclusions:** In this non-randomized comparison, alteplase treatment in patients without an identifiable vessel occlusion did not result in higher rates of favorable outcome compared to untreated. However, treated patients displayed less favorable baseline prognostic factors than the untreated group. Further studies may be required to confirm this data.

## Introduction

Alteplase has been shown to be an effective therapy for ischemic stroke patients when administered within 4.5 h of symptom onset (1–4). However, up to 39% of patients presenting with clinical symptoms with an ischemic stroke have no identifiable vessel occlusion on baseline computed tomographic (CT) angiography (CTA) (5, 6). There are several possible reasons which may result in patients presenting to hospital with the clinical symptoms suggestive of a stroke but with no identifiable vessel occlusion on CTA. These include spontaneous recanalization of a recent occlusion such that no occlusion exists at the time of scanning despite one having been previously present, small and/or distal occlusion beyond the resolution of CTA, hemodynamic infarction without vessel occlusion or a non-stroke cause of an acute focal neurological deficit (7).

The efficacy of alteplase to treat patients without an identifiable vessel occlusion on baseline vascular imaging is controversial (6, 8–11), with some studies demonstrating that such patients may have no better clinical outcome after alteplase (6, 8, 9). An individual patient meta-analysis of alteplase-treated patients suggests the sub-group of patients with mild baseline stroke severity do have a higher odds of favorable outcome after alteplase compared to placebo-treated patients (3). However, the placebo-controlled trials included in the meta-analysis were performed without imaging based patient selection, instead of using only a noncontrast computed tomography (NCCT), meaning that the vessel occlusion status in these patients with mild symptoms is unknown (12). Patients without a baseline occlusion or a perfusion lesion are now often excluded from trials of reperfusion therapy, as identifying a reperfusion therapy “target” improves the likelihood of detecting clinical changes between study groups (13, 14). This is due, in part, to the poor natural history of stroke in patients with an identifiable large vessel occlusion who do not undergo recanalization. Conversely, the natural history of patients without an identifiable vessel occlusion may be very good, and as such there is a ceiling effect for reperfusion therapy in these patients where a high proportion have an excellent clinical outcome without intervention.

Whilst multimodal CT is a powerful tool to predict the clinical outcomes of patients after alteplase therapy (15–17), previous work has also identified subgroups of patients who do not benefit from alteplase or where outcomes may be unfavorable (18, 19). In the present study, we hypothesized that non-lacunar ischemic stroke patients with no visible baseline vessel occlusion on CTA will not show higher rates of excellent clinical outcome [modified Rankin Scale (mRS) 0–1] at 90 days after alteplase therapy when compared to untreated patients taken from a large observational database.

## Patients and methods

### Patients

Ischemic stroke patients presenting to hospital within 12 h of symptom onset were prospectively recruited to the International Stroke Perfusion Imaging Registry (INSPIRE) in participating centers. Contributing to this cohort were seven hospitals across Australia, China, Canada between 2011 and 2014. Patients underwent pre-treatment multimodal CT imaging, including NCCT, CT perfusion (CTP), CT angiography, and follow-up magnetic resonance imaging (MRI) performed at 24-h post stroke. The National Institutes of Health Stroke Scale (NIHSS) was used at baseline to assess clinical severity. Intravenous thrombolysis was administered to patients according to local guidelines and the clinical judgment of the treating physician. For the current study, we restricted our analyses to patients potentially eligible for thrombolysis therapy who presented to the hospital within 4.5 h of onset. In addition to standard clinical and NCCT criteria all patients had multimodal CT prior to treatment, and this information was used by the local treating neurologists as decision assistance at the individual patient level for alteplase eligibility (19). Based on standard NCCT criteria, patients were not eligible for alteplase treatment if they had an intracranial hemorrhage or extensive early ischemic change. In addition, an unfavorable pattern on baseline CTP: small or no identifiable perfusion lesion, a large ischemic core, a lack of perfusion lesion-core mismatch (18, 19) or lack of a vessel occlusion along with perfusion lesion size and clinical characteristics would have been taken into consideration by the treating neurologist in determining a decision to administer alteplase. Thus, in some patients, alteplase treatment may have been withheld at the attending neurologists' discretion based on clinical and radiological characteristics.

The mRS assessed the degree of dependence and disability at 90 days after stroke. Endovascular therapy was not available at all the study centers during INSPIRE recruitment between 2011-2014. The INSPIRE study was approved by the Hunter New England Health District ethics committees in accordance with Australian NHMRC guidelines. All patients in the registry provided written consent in accordance with the Declaration of Helsinki. For this study, patients within the INSPIRE registry were required to have complete baseline clinical characteristics, including baseline NIHSS score and stroke onset time, baseline CTP and CTA imaging, and 3-month mRS score. The INSPIRE registry did not include patients who had a Transient Ischemic Attack, if the patient had a brief episode of neurologic dysfunction, and had no acute infarction on the follow-up imaging (20).

### Baseline imaging

The baseline CT imaging included brain NCCT, CTP, and CTA, gained with different CT scanners (64-, 128-, 256-, or 320- detectors, with Toshiba, Siemens, GE, or Philips scanners). Axial coverage ranged from 40 to 160 mm. After CT perfusion, a CTA was acquired from the aortic arch to vertex (12). The details of each scanner are summarized in Supplementary Table 1.

### Follow-up imaging

All the treated and non-treated patients underwent a magnetic resonance imaging (MRI), using 1.5 Tesla or 3 Tesla scanner between 24 and 48 h post-stroke. The MRI protocol included an axial gradient-echo T2^*^-weighted series, diffusion-weighted image (DWI), perfusion-weighted image (PWI), MR time of flight angiography, as well as fluid-attenuated inversion recovery imaging. A follow-up NCCT and CTP was performed using the above protocols when MR was not available, or the patient had contraindications to MRI.

### Image post-processing and classification of patients

Baseline arterial occlusion status was assessed visually on CTA by three stroke neurologists. All baseline CTA scans were analyzed using maximum intensity projection (MIP) technique in the core laboratory for occlusion site and severity using the thrombolysis in cerebral infarction (TICI) grading system. We classified baseline vessel occlusion status as either (i) normal = TICI 3, (ii) partial = TICI 2a or 2b, or (iii) complete = TICI 1 or 0.

The baseline CTP images were analyzed by the treating clinician before treatment decision, however, the results were not recorded. Therefore, the research team analyzed the baseline and follow-up CTP images retrospectively. Both clinician and the research team used the same commercial software, MIStar (Apollo Medical Imaging Technology, Melbourne, Australia) (21). The threshold for perfusion lesion was defined as a relative delay time (DT) >3 s, and the ischemic core was defined as relative cerebral blood flow (rCBF) <30% within the perfusion lesion (22). Reperfusion was defined as a reduction of the acute perfusion lesion of >80% from the acute to the 24-h perfusion imaging perfusion lesion of zero. Hemorrhagic transformation (HT) was classified based on morphological appearance on follow-up imaging (23) as either hemorrhagic infarction type 1 or 2 (HI1: small petechiae, HI2: more confluent petechiae) or parenchymal hematoma type 1 or 2 (PH1: 30% of the infarcted area with mild space-occupying effect, PH2: 30% of the infarcted area with significant space-occupying effect), and symptomatic intracerebral hemorrhage (sICH) was defined as hemorrhage with NIHSS change ≥4 from baseline to 24-h or leading to death by stroke neurologists blinded to treatment and pre-treatment imaging information.

### Statistical analysis

Statistical analyses were programmed using Stata (v13.0; StataCorp LP, College Station, TX). The aim of this study was to examine the clinical and imaging outcomes of alteplase-eligible patients without a vessel occlusion who were treated with alteplase compared to those who had not been treated. Patients were divided into two groups based on their baseline vessel patency status: no visible vessel occlusion (normal) or visible vessel occlusion (including partial and complete occlusion). Patients without an occlusion were further divided into two subgroups based on the volume of perfusion lesion: these were patients with perfusion lesions <15 mL and those with perfusion lesions >15 mL. The baseline clinical and imaging variables between treated and untreated patients in the no-occlusion group were compared using a two-sample *t*-test, Wilcoxon signed-rank sum test, Kruskal-Wallis, and Fisher's exact tests, where applicable. Additionally, we used Wilcoxon rank-sum test to assess the differences of the NIHSS improvement between treatment groups in patients without a vessel occlusion, and then in patients with a vessel occlusion.

Next, we used inverse propensity score weighting (IPSW) to adjust the baseline bias of variables between the two treatment groups, using this method could enforce the model to use a balanced dataset by gaining the different amount of information from each patient. The IPSW method has three steps. First, we used a logistic regression model to generate a propensity score for each patient, which provides the probability of a patient receiving alteplase treatment according to their baseline characteristics. The outcome of this propensity score generating model was treatment with alteplase (binary outcome, treated vs. untreated), and we used age, baseline NIHSS, onset time to imaging, baseline volume of perfusion lesion, and center (John Hunter Hospital vs. other hospitals) to control the model for confounding. Second, we calculated the inverse probability of receiving thrombolysis or not from the propensity score. Last, we used the inverse probabilities as weights in the final logistic regression model for each patient, to measure the relative risk for treatment with alteplase in patients without a vessel occlusion. We calculated the absolute standardized difference for each baseline variables before and after propensity score matching, to check the baseline bias of treated and untreated groups. The outcomes in the final logistic regression model were: Excellent (mRS 0–1 vs. 2–6), Good (mRS 0–2 vs. 3–6), and Poor (mRS 5–6 vs. 0–4). The propensity analysis was applied to patients who had visible baseline vessel occlusion, patients who had no visible baseline vessel occlusion, as well as the two no vessel occlusion subgroups, perfusion lesions <15 mL and perfusion lesions >15 mL.

Furthermore, an additional sensitivity analysis was performed for patients who had no visible vessel occlusion using regression adjustment with the propensity score. Multivariable logistic regression was performed to assess the effect of alteplase treatment and baseline vessel occlusion status, covariates included age, baseline NIHSS score, onset time to imaging, baseline volume of perfusion lesion, center, and the interaction term of baseline vessel occlusion status and treatment.

## Results

At the time of this analysis, the INSPIRE registry contained 1,569 patients. Two hundred and ninety two patients were not eligible for analysis: 134 patients were excluded because they were given alteplase outside the 4.5-h time window, 28 patients had poor quality baseline imaging due to motion artifacts, 89 untreated patients had documented clinical contraindications to thrombolysis (e.g., rapid clinical improvement, premorbid disability, or major early ischemic change on NCCT), and 41 patients with lacunar strokes on follow-up MRI were excluded. Of the remaining 1,277 patients who were clinically eligible for thrombolysis, 306 (24%) had no vessel occlusion at baseline (TICI 3) compared to 971 (76%) who had a vessel occlusion (TICI 0-2b). Of the patients without a baseline occlusion and eligible for thrombolysis, 141 (46%) were treated with alteplase, and 165 (54%) were not treated. In comparison, of those patients with a baseline occlusion, 732 (75%) received alteplase, and 239 (25%) did not treat.

**Figure 1:**
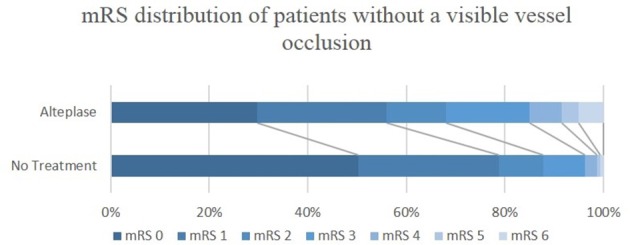
mRS distribution of ischemic stroke patients without a visible vessel occlusion, who were or were not treated with alteplase. mRS, modified Rankin Scale.

Of the patients without a baseline occlusion, alteplase treated patients had higher median baseline NIHSS score (8, interquartile range (IQR) 5–10 for treated vs. 6, IQR 4–8 for untreated patients, *P* < 0.001) and larger median perfusion lesions (5.6 mL, IQR 1.3–17.7 mL for treated vs. 2.6 mL, IQR 0–6.7 mL for untreated patients, *P* < 0.001, Table 1). The baseline imbalances between alteplase treated and untreated groups were reduced after propensity score matching (Supplementary Tables 2, 3). Patients without a vessel occlusion at baseline who were treated with alteplase had reduced odds of achieving an excellent or good 90-day clinical outcome than patients who were not treated [mRS 0–1, alteplase 56%, untreated 79%, odds ratio (OR) 0.36, 95% confidence interval (CI) 0.22–0.59, *P* < 0.001; mRS 0–2, alteplase 68%, untreated 88%, OR 0.31, 95% CI 0.17–0.56, *P* < 0.001, Table 2, Figure 1] even following propensity matching (mRS 0–1, OR 0.42, 95% CI 0.24–0.75, *P* = 0.003; mRS 0–2, OR 0.41, 95% CI 0.21–0.81, *P* = 0.010, Table 2). Both sensitivity analysis and results from multivariable regression with an interaction term of baseline vessel occlusion status and treatment yielded similar results (Supplementary Tables 4, 5). Additionally, alteplase treated patients with no vessel occlusion had an increased odd of poor clinical outcome than untreated patients (mRS 5–6, alteplase 9%, untreated 1%, OR 7.58, 95% CI 1.67–34.48, *P* = 0.009), however this was no longer significant following propensity matching (mRS 5–6, OR 3.09, 95% CI 0.50–19.01, *P* = 0.223, Table 2). Lastly, all the patients who had no visible baseline occlusion and 24-h perfusion imaging available had complete reperfusion (*n* = 69, Table 1).

**Table 1:** Clinical and imaging characteristics of patients with and without a visible baseline occlusion who were also clinically eligible for alteplase therapy.

		**Visible baseline occlusion**		***P-*value**	**No visible baseline occlusion**		***P-*value**
	**Total**	**Treated**	**Untreated**		**Treated**	**Untreated**	
	***N* = 1277**	***N* = 732**	***N* = 239**		***N* = 141**	***N* = 165**	
Age, mean (SD), y	70.7 (13.9)	72.0 (12.5)	70.4 (16.0)	0.109	68.1(14.4)	67.5 (15.2)	0.714
NIHSS, median (IQR)	12 (8 to 17)	14 (10 to 18)	13 (10 to 17)	0.015	8 (5 to 10)	6 (4 to 8)	< 0.001
Onset to imaging, median (IQR), min	120 (80 to 160)	121 (91 to 157)	150 (81 to 163)	0.120	89.1 (46.5 to 139)	98.5 (58 to 159)	0.099
Perfusion lesion volume, median (IQR), mL	61.7 (15.5 to 129.2)	97.0 (52.5 to 148.0)	60.1 (33.0 to 137.1)	<0.001	5.6 (1.3 to 17.7)	2.6 (0 to 6.7)	<0.001
Ischemic core volume, median (IQR), mL	12.9 (2.7 to 37.0)	19.8 (8.2 to 54.1)	16.9 (8.3 to 51.8)	0.418	1.8 (0.1 to 6.4)	0.4 (0 to 1.9)	<0.001
Reperfusion, *n* (%)	332 (54.4)	280 (49.8)	55 (44.7)	0.326	33 (100)	36 (100)	–
NIHSS improvement, median (IQR)	–	4 (24 to −17)	3 (15 to −6)	0.008	4 (13 to −3)	4 (17 to −8)	0.913
Any HT, *n*	172	146 (19.9)	12 (5)	<0.001	14 (9.9)	0 (0)	<0.001
HI1, *n* (%)	58	49 (6.7)	3 (1.3)	<0.001	6 (4.3)	0 (0)	0.009
HI2, *n* (%)	39	34 (4.6)	4 (1.7)	0.052	1 (0.7)	0 (0)	0.461
PH1, *n* (%)	38	34 (4.6)	1 (0.4)	0.001	3 (2.1)	0 (0)	0.097
PH2, *n* (%)	37	29 (4.0)	4 (1.7)	0.102	4 (2.8)	0 (0)	0.044
sICH, *n* (%)	28	22 (3.0)	3 (1.3)	0.163	3 (2.1)	0 (0)	0.097

*Values are presented as means with standard deviation (SD), medians with inter-quartile range (IQR), or number (%). NIHSS, National Institutes of Health Stroke Scale; HT, hemorrhagic transformation; HI, hemorrhagic infarction; PH, parenchymal hematoma; sICH, symptomatic intracerebral hemorrhage*.

**Table 2:** The effect of alteplase treatment in patients without a visible vessel occlusion (TICI 3).

**Outcome (% treated/% untreated)**	**Without propensity analysis**		***P*-value**	**Propensity analysis**		***P*-value**
	**OR**	**95% CI**		**OR**	**95% CI**	
Excellent (56/79)	0.36	0.22 to 0.59	<0.001	0.42	0.24 to 0.75	0.003
Good (68/88)	0.31	0.17 to 0.56	<0.001	0.41	0.21 to 0.81	0.010
Poor (9/1)	7.58	1.67 to 34.48	0.009	3.09	0.50 to 19.01	0.223

*The results are from logistic regression models without and with propensity analysis. OR, odds ratio; CI, confidence interval*.

A total of 14 patients without a vessel occlusion had a hemorrhagic transformation on follow-up imaging, all of whom were treated with alteplase (9.9% of all alteplase treated non-occlusion patients, 6 HI1, 1 HI1, 3 PH1, and 4 PH2). The rate of HI1 and PH2 was significantly higher in alteplase treated patients without a vessel occlusion compared to untreated (HI1, alteplase 4.3%, untreated 0%, *P* = 0.009; PH2, alteplase 2.8%, untreated 0%, *P* = 0.044, Table 1). There were 3 non-occlusion patients with sICH, all of whom were treated (sICH, alteplase 2.1%, untreated 0%, *P* = 0.097). Of the 14 patients with any HT, 9 of them had baseline perfusion deficits <15 mL, and 2 of the 3 patients with sICH had <15 mL perfusion deficits at baseline.

After propensity score adjustment, alteplase treated patients without a vessel occlusion and perfusion lesions <15 mL had lower rates of excellent or good outcome compared to untreated patients (mRS 0–1, alteplase 63%, untreated 81%, OR 0.51, 95% CI 0.27–0.94, *P* = 0.031; mRS 0–2, alteplase 72%, untreated 91%, OR 0.40, 95% CI 0.19–0.86, *P* = 0.018, Table 3). Additionally, the alteplase treated patients without a baseline vessel occlusion and perfusion lesions <15 mL had more frequently had poor outcomes compared to untreated patients (mRS 5–6, alteplase 6%, untreated 0%, Table 3). Similarly, the rate of excellent or good outcome was not higher in alteplase treated patients without a vessel occlusion and perfusion lesions >15 mL (mRS 0–1, alteplase 38%, untreated 53%, OR 0.17, 95% CI 0.04–0.73, *P* = 0.017; mRS 0–2, alteplase 58%, untreated 60%, OR 0.34, 95% CI 0.08–1.49, *P* = 0.151), however, the rate of poor outcome did not differ between groups (mRS 5–6, alteplase 15%, untreated 13%, OR 1.48, 95% CI 0.18–11.90, *P* = 0.715, Table 3).

**Table 3:** The effect of alteplase treatment in patients without a vessel occlusion grouped by the volume of perfusion lesions, logistic regression results with propensity analysis.

**Outcome**	**Perfusion lesions**<**15 mL**			***P-*value**	**Perfusion lesion** >**15 mL**			***P-*value**
	**% treated/% untreated**	**OR**	**95% CI**		**% treated/% untreated**	**OR**	**95% CI**	
Excellent	63/81	0.51	0.27–0.94	0.031	38/53	0.17	0.04–0.73	0.017
Good	72/91	0.40	0.19–0.86	0.018	58/60	0.34	0.08–1.49	0.151
Poor	6/0	–	–	–	15/13	1.48	0.18–11.90	0.715

*OR, odds ratio; CI, confidence interval*.

**Figure 2:**
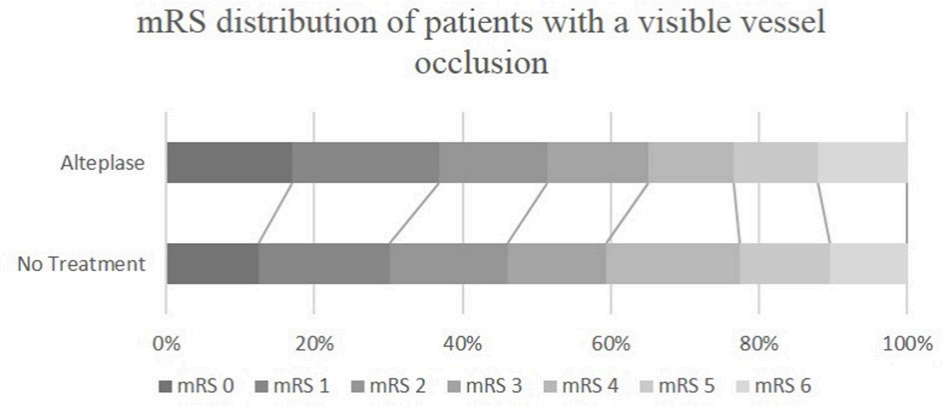
mRS distribution of ischemic stroke patients with a visible vessel occlusion, who were or were not treated with alteplase. mRS, modified Rankin Scale.

As expected, patients with a vessel occlusion at baseline had a trend toward higher odds of achieving an excellent or a good outcome at 90-day when treated with alteplase (mRS 0-1, alteplase 37%, untreated 30%, OR 1.36, 95% CI 0.99–1.86, *P* = 0.058; mRS 0–2, alteplase 52%, untreated 46%, OR 1.25, 95% CI 0.93–1.67, *P* = 0.142, Table 4, Figure 2). This became highly significant after propensity analysis (mRS 0–1, OR 1.84, 95% CI 1.32–2.58, *P* < 0.001; mRS 0–2, OR 1.71, 95% CI 1.25–2.32, *P* = 0.001, Table 4).

**Table 4:** The effect of alteplase treatment in patients with a visible vessel occlusion (TICI 0-2b).

**Outcome (% treated/% untreated)**	**Without propensity analysis**		***P*-value**	**Propensity analysis**		***P-*value**
	**OR**	**95% CI**		**OR**	**95% CI**	
Excellent (37/30)	1.36	0.99–1.86	0.058	1.84	1.32–2.58	<0.001
Good (52/46)	1.25	0.93–1.67	0.142	1.71	1.25–2.32	0.001
Poor (24/23)	1.05	0.74–1.49	0.774	0.74	0.51–1.06	0.097

*The results are from logistic regression models without and with propensity analysis. OR, odds ratio; CI, confidence interval*.

## Discussion

We observed that patients without a baseline vessel occlusion who, based on standard clinical and NCCT criteria (and treating neurologists prior knowledge of CTA and CTP profiles), receive alteplase therapy, showed significantly lower odds of favorable clinical outcome compared to a population of patients not treated with alteplase where the treating neurologist also was aware of the patients CTA and CTP profiles. The natural history of patients without a vessel occlusion, where the decision was made by the clinical team not to treat resulted in 79% of untreated patients being functionally independent at 90 days. Importantly, however, this still leaves 21% of patients with disability at 90 days indicating that some form of treatment is warranted. However, by comparison, patients without a vessel occlusion who were treated with alteplase had a lower rate of excellent clinical outcome (56%) at 90 days. Importantly, in patients without a baseline vessel occlusion who were treated with alteplase, the rate of hemorrhage was significantly higher than those who were not treated. Therefore, the risk of hemorrhage after alteplase treatment might be the reason for the observed higher rate of poor outcome in treated patients. Our results are consistent with previous observational studies, suggesting that treatment of patients with no baseline occlusion with alteplase are less likely to be of benefit (6, 8, 9), while exposing patients to the risk of hemorrhage (18). Taken together, the lower rate of excellent outcome and increased risk of hemorrhage as a result of alteplase therapy in patients without a baseline vessel occlusion indicate that a safer thrombolytic is required which would target the 21% of patients who were not disability-free at 90 days.

Patients included in the study had milder stroke severity at baseline compared to studies designed to test the efficacy of alteplase on ischemic stroke patients (11). This is a critical point because it highlights a difference between an observational study and the clinical trials that validated the use of thrombolysis. Our study includes many patients who may have been considered too mild to be enrolled in clinical trials designed to measure alteplase effectiveness. This milder stroke population within INSPIRE has contributed to our ability to show a significant difference between our study populations, both with and without propensity analysis. The study included a large proportion of patients without a vessel occlusion and perfusion lesion <15 mL (82%), a subgroup previously reported to gain no net benefit from alteplase therapy (18). We have noted a similar lack of benefit in the subgroup of patients without a vessel occlusion and perfusion lesion >15 mL, suggesting patients without a vessel occlusion are less likely to be of benefit from alteplase treatment, regardless of the volume of baseline perfusion lesion. However, it is important to note the relatively small numbers of patients with perfusion lesion volumes above 15 mL and the possibility that some patients included in this study without a baseline occlusion may have spontaneously recanalized before baseline imaging. Potentially the latter are patients with a relatively the high risk of hemorrhage with alteplase therapy. This notion is supported by the data that all patients without a baseline occlusion showed reperfusion at 24 h, regardless of alteplase therapy.

Some study limitations require acknowledgment. (1) Given the observational design, there were significant differences between baseline characteristics of patients treated and untreated with alteplase across all the study groups in terms of their baseline clinical severity which may have skewed the results toward favoring the untreated patient group even after propensity adjustment. (2) The differences in baseline characteristics between the treated and untreated patients without a baseline vessel occlusion will likely have contributed the worse outcomes in the treated patients, however measuring any clinical improvement in this cohort will be difficult due to an excellent natural history. (3) It is challenging to assess the M3 and M4 occlusions, however, we found patients who had no vessel occlusion, had minimal perfusion lesion volumes which were not in the distal territories, were reperfused on 24-h imaging and had minimal DWI lesion volumes. This suggests that patients in this study were more likely to have spontaneously recanalized, since there was no acute radiological treatment target and minimal clinical deficit at 24 h. (4) It appears the INSPIRE site neurologists were reasonably adept at identifying patients with a favorable natural history and thus withholding treatment even when they appeared to fullfil standard clinical criteria for thrombolysis. However, due to the database design, we have not captured all the information that led to the treating neurologists avoiding thrombolysis in such patients and unmeasured confounding variables are also possible influences. Indeed, it may be difficult to measure some influences on clinician behaviors such as perceptions of patient frailty or other impressions related to an end of the bed “gestalt” assessment of the patient and their imaging. (5) INSPIRE is a large study and sites are strongly encouraged to enroll consecutive patients, the need for pre-treatment multimodal CT and follow-up MR, along with clinical data from several time-points means that not all treated or untreated patients at every center are included, which may bias our sample against generalizability. (6) There may be variables presented which INSPIRE has not captured which may be of relevance to the results, such as patient frailty or undocumented comorbidities. (7) We understand that the mRS at 90-day were assessed by different clinicians across sites, which may influence the interpretation of our data. Therefore, we dichotomized the mRS outcomes to Excellent (mRS 0–1 vs. 2–6), Good (mRS 0–2 vs. 3–6), and Poor (mRS 5–6 vs. 0–4), which may potentially reduce the influence of different jurisdictions on our data compared to treating the mRS outcome as an ordinal variable. 8) This study assessed only a single time point with perfusion imaging, repeated measures may be an ideal method to capture dynamic changes in cerebral blood flow to assess if patients had spontaneous recanalization. (9) The data presented is hypothesis generating and cannot replace a randomized clinical trial.

## Conclusions

This study suggests that patients without a vessel occlusion may be less likely to have clinical improvement due to alteplase therapy. This may very likely represent a treatment selection bias, however and there is currently no evidence that alteplase should be withheld in this group of patients. An alternative thrombolytic agent may be ideal in patients without a baseline vessel occlusion in order to reduce the hemorrhage rate and reduce the rate of long-term disability in patients which a clinical belief that treatment is required.

## Author contributions

HT, MP, CL, AB contributed to the concept and design of the study, analysis of data, and drafting the manuscript and figures. XC, RA, NS, TK, BO, KB, LL, JZ, QD, CC contributed acquisition and analysis of data, drafting the manuscript and figures.

### Conflict of interest statement

The authors declare that the research was conducted in the absence of any commercial or financial relationships that could be construed as a potential conflict of interest.
