# Supplementary material for: Intravenous Thrombolysis May Not Improve Clinical Outcome of Acute Ischemic Stroke Patients Without a Baseline Vessel Occlusion
Source: Front Neurol. 2018 Jun 6;9:405. doi: 10.3389/fneur.2018.00405 (PMC5997810; doi:10.3389/fneur.2018.00405)
Supplement: Supplementary file 1 [file Table_1.docx]

**Supplementary Table 1.** CTP acquisition protocols for the seven INSPIRE sites involved in this study

| **Site** | **CT Scanner** | **Acquisitions** | **Contrast** | **Coverage** |
| --- | --- | --- | --- | --- |
| John Hunter Hospital, Australia | Toshiba Aquilion one  – 320 detectors | 19 acquisitions in 60 seconds | 40 ml of contrast (Ultravist 370) injected at 6 mL/s, followed by 30 mL of saline | 160 mm |
| Huashan Hospital, China | Philips Brilliance iCT  – 128 detectors | 23 acquisitions in 60 seconds | 40 mL of contrast (Ultravist 370) injected at 5 mL/s, followennyd by 20 mL saline | 125 mm |
| Sunnybrook Medical Center, Canada | GE Healthcare lightspeed  – 64 detectors | 51 acquisitions in 135 seconds | 0.7 mL/kg of iodinated contrast agent up to a maximum 90 mL (Omnipaque 300 mg iodine/mL) injected at 2-4 mL/seconds | 40 mm |
| Royal Adelaide Hospital | Siemens Somatom Definition AS+  – 128 detectors | 19 acquisitions in 60 seconds | 40 mL of contrast (Ultravist 370) at 6 mL/s, followed by 30 mL of saline | 96 mm |
| Gosford Hospital, Australia | GE Healthcare lightspeed  – 64 detectors | 2 slabs: 19 acquisitions in 54 seconds each slab | 45 mL of contrast (Ultravist 370) at 6 mL/s | 80 mm |
| University of Alberta, Canada | Siemens Somatom Definition AS+  – 256 detectors | 51 acquisitions in 135 seconds | 40 mL of contrast (Ultravist 370) at 6 mL/s | 100 mm |
| Baotou Central Hospital, China | Siemmens Somatom Definition Flash  – 256 detectors | 51 acquisitions in 135 seconds | 40 mL of contrast (Ultravist 350) at 5 mL/s | 100 mm |
