# Supplementary material for: Intravenous Thrombolysis May Not Improve Clinical Outcome of Acute Ischemic Stroke Patients Without a Baseline Vessel Occlusion
Source: Front Neurol. 2018 Jun 6;9:405. doi: 10.3389/fneur.2018.00405 (PMC5997810; doi:10.3389/fneur.2018.00405)
Supplement: Supplementary file 2 [file Table_2.docx]

**Supplementary Table 2.** Baseline characteristics of patients without a vessel occlusion, before and after propensity score matching

| **Variable** | **Unmatched/**  **Matched** | **Mean/Percentage** | | **Absolute standardized difference** | ***P*-value** |
| --- | --- | --- | --- | --- | --- |
|  |  | Treated | Untreated |  |  |
| Age | U | 68.09 | 67.47 | 4.2 | 0.714 |
|  | M | 68.09 | 67.15 | 6.3 | 0.617 |
| Baseline NIHSS | U | 8.69 | 6.61 | 42.9 | <0.001 |
|  | M | 8.69 | 7.95 | 15.3 | 0.248 |
| Baseline perfusion lesion (mL) | U | 13.91 | 6.59 | 41.6 | <0.001 |
|  | M | 13.91 | 13.41 | 2.8 | 0.809 |
| Baseline ischemic core (mL) | U | 6.07 | 2.74 | 31.8 | 0.006 |
|  | M | 6.07 | 5.92 | 1.5 | 0.904 |
| Center (JHH) | U | 22% | 46% | 52.4 | <0.001 |
|  | M | 22% | 19% | 6.2 | 0.557 |

After propensity score matching, treated and untreated patients without a vessel occlusion have smaller absolute standardized difference at baseline, and the difference between the two treatment groups are not statistically significant. JHH: John Hunter Hospital. Center dichotomized to John Hunter Hospital versus other hospitals.
