# Supplementary material for: Intravenous Thrombolysis May Not Improve Clinical Outcome of Acute Ischemic Stroke Patients Without a Baseline Vessel Occlusion
Source: Front Neurol. 2018 Jun 6;9:405. doi: 10.3389/fneur.2018.00405 (PMC5997810; doi:10.3389/fneur.2018.00405)
Supplement: Supplementary file 3 [file Table_3.docx]

**Supplementary Table 3.** Baseline characteristics of patients with a vessel occlusion, before and after propensity score matching

| **Variable** | **Unmatched/**  **Matched** | **Mean/Percentage** | | **Absolute standardized difference** | ***P*-value** |
| --- | --- | --- | --- | --- | --- |
|  |  | Treated | Untreated |  |  |
| Age | U | 72.03 | 70.42 | 11.2 | 0.109 |
|  | M | 72.03 | 71.45 | 4 | 0.381 |
| Baseline NIHSS | U | 14.139 | 13.11 | 19.2 | 0.012 |
|  | M | 14.139 | 14.4 | 5.1 | 0.329 |
| Baseline perfusion lesion (mL) | U | 110.7 | 87.99 | 27.1 | <0.001 |
|  | M | 110.7 | 112.27 | 1.9 | 0.731 |
| Baseline ischemic core (mL) | U | 41.03 | 32.13 | 17.8 | 0.033 |
|  | M | 41.03 | 40.25 | 1.6 | 0.777 |
| Center (JHH) | U | 41.80% | 62.70% | 42.9 | <0.001 |
|  | M | 41.80% | 43% | 2.5 | 0.634 |

After propensity score matching, treated and untreated patients with a vessel occlusion have smaller absolute standardized difference, and the difference between the two treatment groups are not statistically significant. JHH: John Hunter Hospital. Center dichotomized to John Hunter Hospital versus other hospitals.
