# Supplementary material for: Intravenous Thrombolysis May Not Improve Clinical Outcome of Acute Ischemic Stroke Patients Without a Baseline Vessel Occlusion
Source: Front Neurol. 2018 Jun 6;9:405. doi: 10.3389/fneur.2018.00405 (PMC5997810; doi:10.3389/fneur.2018.00405)
Supplement: Supplementary file 4 [file Table_4.DOCX]

**Supplementary Table 4.** Sensitivity analysis after regression adjustment with propensity score (patients who had no visible vessel occlusion)

| Outcome  (% treated / % untreated) | OR | | 95% CI | *P*-value |
| --- | --- | --- | --- | --- |
| Excellent (56 / 79) | 0.40 | 0.22-0.73 | | 0.003 |
| Good (68 / 88) | 0.40 | 0.20-0.82 | | 0.013 |
| Poor (9 / 1) | 13.98 | 1.83-106.99 | | 0.009 |

OR indicates odds ratio; CI, confidence interval
