# Supplementary material for: Intravenous Thrombolysis May Not Improve Clinical Outcome of Acute Ischemic Stroke Patients Without a Baseline Vessel Occlusion
Source: Front Neurol. 2018 Jun 6;9:405. doi: 10.3389/fneur.2018.00405 (PMC5997810; doi:10.3389/fneur.2018.00405)
Supplement: Supplementary file 5 [file Table_5.docx]

**Supplementary Table 5.** Effect of thrombolysis and vessel occlusion status in multivariable logistic regression

| Outcomes | Alteplase treated patients | OR | 95% CI | *P-*value | R-squared | AUC (95% CI) |
| --- | --- | --- | --- | --- | --- | --- |
| Excellent | No occlusion | 0.38 | 0.22-0.66 | 0.001 | 0.191 | 0.80 (0.77-0.82) |
|  | occlusion | 4.89 | 2.56-0.34 | <0.001 |  |  |
| Good | No occlusion | 0.38 | 0.20-0.70 | 0.002 | 0.187 | 0.79 (0.77-0.82) |
|  | occlusion | 4.50 | 2.22-9.11 | <0.001 |  |  |
| Poor | No occlusion | 9.10 | 1.14-72.97 | 0.038 | 0.263 | 0.86 (0.83-0.88) |
|  | occlusion | 0.08 | 0.01-0.67 | 0.02 |  |  |

The multivariable logistic regression included the interaction term of baseline vessel occlusion status and thrombolysis, other covariates were age, baseline NIHSS score, onset time to imaging, baseline volume of perfusion lesion, center (John Hunter Hospital versus other). OR indicates odds ratio; CI, confidence interval, AUC: area under the curve.
